# Typhoid Fever in New York

**Published:** 1884-02

**Authors:** 


### TYPHOID FEVER IN NEW YORK.

The death rate in this city so far this year has been unusually low, and the prospects are that the record for the year will correspond. The greatest danger is from the increasing prevalence of typhoid fever. The impression that the fever infection results only from contamination by ingestion is gradually giving place to the belief that a lodgment may also be effected in the air passages. In conjunction with the Board of Health physicians can do much toward stopping the advance of the disease by enforcing the immediate disinfection of typhoid fever excreta. The Board has issued circulars giving directions for the best means of accomplishing this object.—*Scientific American.*

THIRSTY CHILDREN.—There is nothing from which infants and children suffer so much as from thirst. They require water, usually, ten times where they get it once. Infants should have a teaspoonful or more of cold water every hour, commencing when they are a week old. Infants often cry so as to disturb every one present. If a sip of water is given to a child who seems to be crying without cause, it will stop instantly in nine cases out of ten. Thirst causes more bad tempers in children than anything else. We speak of anything being "as free as water." Let the children share this freedom, and they will be better and healthier for it.

WE desire to call the attention of our readers to the advertisement of Knight Brothers, Solicitors of Patents, Washington, D. C. We have done business with this firm for fifteen years, and can confidently recommend them to those having business with the patent office, where skill and experience are of the utmost importance. It is well known that a slight mistake or omission may destroy the value of a patent. A firm with the experience of Knight Brothers are not likely to make mistakes.

CHILDREN always suffer more or less from costiveness of the bowels. Laxatives relieve the trouble temporarily, but result after a while in increasing the trouble. The use of NELATON'S SUPPOSITORIES FOR CHILDREN (see advertisement on page vii.) will promptly and permanently relieve costiveness, and will also allay the itching from which children often suffer, and destroy pin worms on which the itching often depends. 50 cents a box, post-paid. Hall & Ruckel, 218 Greenwich St., New York.

